# Age-dependent differences in anaphylaxis: elderly population

**DOI:** 10.1097/ACI.0000000000001091

**Published:** 2025-08-11

**Authors:** Alessandro Barone, Francesca Nicoletta, Martina Ottoni, Erminia Ridolo

**Affiliations:** a Allergy and Clinical Immunology, Medicine and Surgery Department, University of Parma, Parma; b Departmental Unit of Allergology, Guglielmo da Saliceto Hospital, Piacenza, Italy

**Keywords:** adrenaline, anaphylaxis, elderly population, food allergy, senescence

## Abstract

**Purpose of review:**

Anaphylaxis in elderly is a little-known topic, despite the worldwide growth of this part of the population. In this review, the main elicitors are discussed, with a particular regard for risk factors, clinical manifestation and management of anaphylaxis in people over 65 years of age.

**Recent findings:**

Available data report age-dependent differences regarding elicitors, cofactors and symptoms of anaphylaxis. In the last years, few studies have focused on anaphylaxis in the elderly, highlighting drugs and insect venom as main triggers.

**Summary:**

Drugs and insect venom represent the main triggers of anaphylaxis in individuals over 65 years of age. In addition, idiopathic anaphylaxis is seen more frequently in adults and older adults, and recent studies show an increasing rate of food-related anaphylaxis in this population.

Elderly patients are at a greater risk of severe or fatal reactions because they often have multiple comorbidities requiring the concomitant use of several drugs. This may complicate anaphylaxis management, leading to poor outcomes, increased hospitalization and higher admission to intensive care unit.

The clinical presentation of anaphylaxis in older adults is most often characterized by cardiovascular symptoms, with syncope as the most frequent one.

The injection of adrenaline is the most important treatment of anaphylaxis at any age, and no absolute contraindications are reported. Despite this, its use still remains suboptimal.

## INTRODUCTION

Anaphylaxis is one of the most severe forms of allergy, representing an acute potentially fatal reaction. The World Allergy Organization Anaphylaxis Guidance 2020 [1] defined anaphylaxis as “a serious systemic hypersensitivity reaction that is usually rapid in onset and may cause death” and stated that “severe anaphylaxis is characterized by potentially life-threatening compromise in airway, breathing and/or circulation, and may occur without typical skin features or circulatory shock being present”. The clinical diagnosis of anaphylaxis can be performed using the diagnostic criteria [1].

Anaphylaxis is typically a multiorgan condition involving a broad range of effector cells including mast cells, basophils, neutrophils, macrophages and platelets. From a mechanistic standpoint, anaphylaxis can be categorized as immunologic, nonimmunologic or idiopathic, with the latter category caused by unidentified allergens or underlying mastocytosis [2]. Immunologic anaphylaxis can be further subcategorized into immunoglobulin E (IgE)-mediated (e.g., food, drugs, and insect stings) and IgE-independent forms, which include immunoglobulin G (IgG)-dependent anaphylaxis (e.g., high molecular weight iron dextran, and infusion of human mAbs, such as infliximab), and complement-mediated (e.g., oversulfated chondroitin sulfate-contaminated heparin and polyethylene glycols) [2]. Mixed reactions involving both IgE and non-IgE mediated pathways can also occur with chemotherapy [2]. Non immunologic anaphylaxis may be caused by direct mediator release from mast cells and basophils (e.g., opioids), physical factors (e.g., exercise, heat, and sunlight/UV radiation), contact system activation (e.g., dialysis membranes) and arachidonic acid metabolism imbalance, as in the case of NSAIDs [2].

Anaphylaxis may occur at any age, and its incidence is rising in Western countries. As life expectancy and the prevalence of older adults becomes higher, the incidence of anaphylactic reactions raises among the geriatric population [3^▪▪^].

Several age-related factors may potentially contribute to anaphylaxis in older people. These include mechanisms related to immune senescence, inflammation, comorbidities and changes in gastrointestinal function as well as micronutrient deficiencies and the use of multiple medications [3^▪▪^,^4^]. 

**Box 1:**
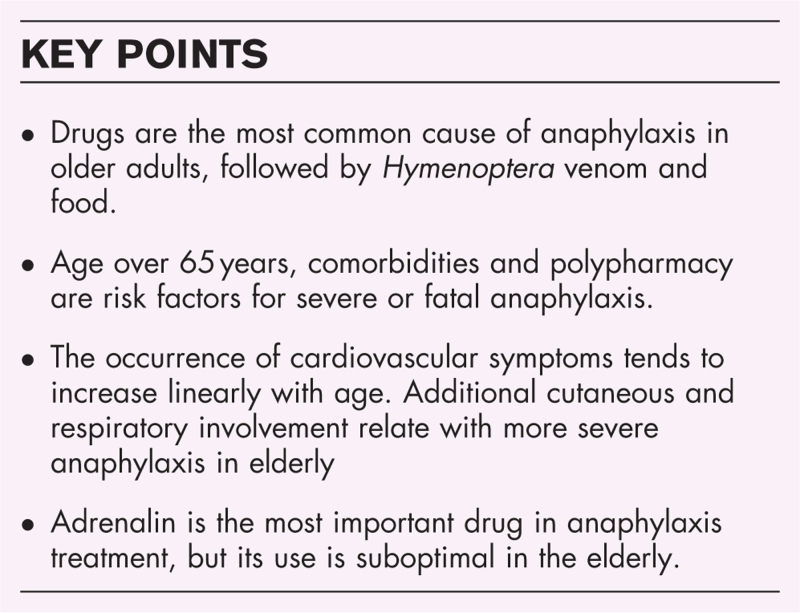
no caption available

**Figure 1:**
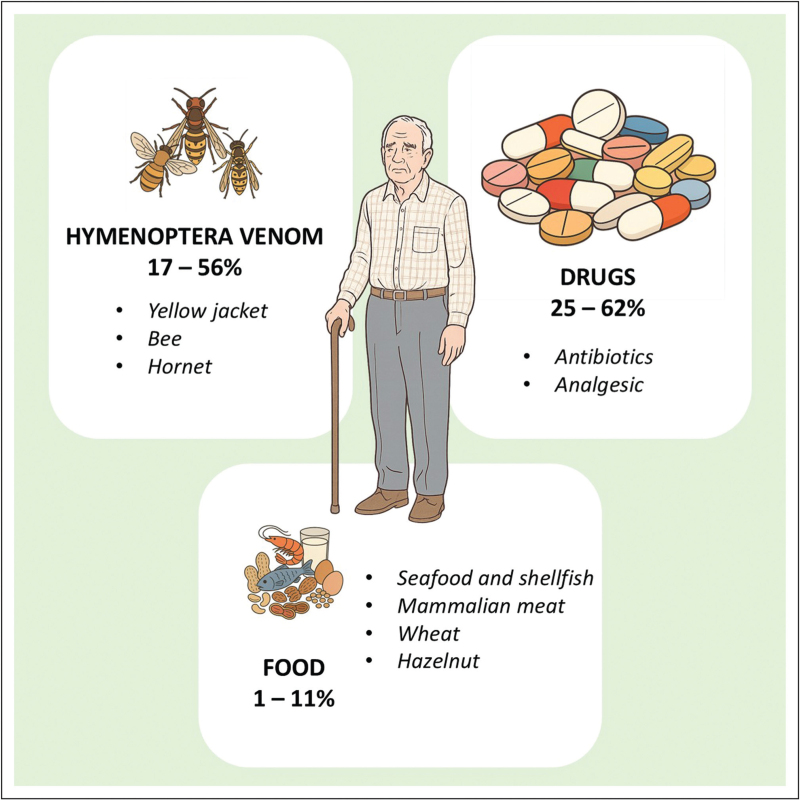
Anaphylaxis in elderly: elicitors. The percentages shown in the figure represent the prevalence of anaphylaxis in older people.

Existing data indicate age-dependent differences regarding elicitors, cofactors and symptoms of anaphylaxis. Overall, elderly patients have a higher risk for severe and fatal reactions [4]. The main elicitors of anaphylaxis are drugs, insect venoms, and food (Fig. 1). While food is the most frequent trigger in children and young adults, anaphylaxis caused by drugs and venom insect are more common in elderly patients [4]. In this regard, it is possible to speculate that these findings might be related to the increased prevalence of cardiovascular diseases leading to limited compensation mechanisms, but also to the use of cardiovascular drugs (i.e., beta-blockers) [4]. Facilitating cofactors like exercise, drugs, alcohol and stress are supposed to reduce the threshold for allergic reactions [4,5]. Recommendations for the emergency treatment of anaphylaxis are similar for all age groups and are supported by current guidelines [1]. Some considerations and adaptations should be made in elderly patients [6]. Although the administration of adrenaline is not contraindicated in anaphylactic patients with known or suspected cardiovascular diseases, it might cause difficulties due to the increasing of coronary blood flow, for example, in patients with acute coronary syndrome. However, current guidelines clearly state that the benefit of adrenaline injection outweighs the risks, even in suspected anaphylaxis [1,5–7].

This review is aimed to focus on the main elicitors of anaphylaxis in geriatric population, with a particular regard to clinical presentation, risk factors and management of the reactions.

## FOOD TRIGGERS

The food allergy is predominantly considered a pediatric condition, but it is increasingly clear that in the last two decades, the prevalence of this condition affects a large number of adults around the world. Food allergy can persist into adulthood among patients diagnosed during childhood, but the development of new-onset allergies later in life is ever more frequently reported [3^▪▪^,^8^].

Overall, the most common food triggers among adults with pediatric-onset allergy include eggs, peanuts and milk [3^▪▪^]. On the other hand, the most prevalent adult-onset food allergies are triggered not only by peanut and milk, but also by fish, shellfish and tree nut [3^▪▪^]. Literature regarding older people is limited, but the most reported food elicitors are shellfish and cow's milk [3^▪▪^,^9^].

It has been recently suggested that patterns of food-induced anaphylaxis (FIA) appear quite different in older people compared to younger groups. Seafood, mammalian meat (often related to alpha-gal sensitization), legumes, fruits and vegetables are the most frequent elicitors of FIA, while anaphylactic reactions to nuts, milk or egg (typical of younger people) have been seldomly reported [10,11^▪^]. Starting from the data of the European Anaphylaxis Registry, Aurich *et al.*[5] reported a prevalence of 11% of FIA in European older adults, with wheat, hazelnut and shellfish being the most frequent elicitors. Although these food triggers are universal, differences might depend on nutritional habits, exposure, geographical and urbanization differences. In particular, it is important to highlight that also elderly patients with seasonal allergic rhinitis may exhibit allergic cross-reactions to plant-derived food allergens sharing a structural similarity to certain pollen proteins. This condition constitutes the so-called pollen-food allergy syndrome (PFAS), which includes the mild form oral allergy syndrome (OAS) and accounts among the most common cases of adult-onset FA. Even though symptoms of OAS are usually limited to oropharyngeal mild reactions, PFAS may present in various forms, characterized by different severity [3^▪▪^]. FIA caused by PFAS are generally low-risk conditions, but it is not always true for elderly due to various reasons, for example, worsened mastication and quality of saliva, atrophic gastritis, use of antiulcer drugs and intestinal inflammation with increased permeability [3^▪▪^,^10^,^12^,^13^]. Unfortunately, available data on this specific topic are still limited.

Different mechanisms underlie the onset of food allergy in adulthood and in the elderly. Aging is accompanied by several physiological and pathological changes that can mask food allergy symptoms or promote them, that is, gastrointestinal alterations, immunosenescence with the transition to a Th2 profile, increased inflammatory status, microbiota alterations and micronutrient deficiency (vitamin D, iron, zinc, etc.). Furthermore, the presence of comorbidities such as chronic pulmonary and cardiovascular diseases, as well as polypharmacy (e.g., beta-blockers and ACE inhibitors) negatively affects the severity of symptoms and the response to treatment in case of FIA [3^▪▪^,^4^].

Cofactors favoring the onset of FIA are typically more common in adults. They include physical exertion, alcohol, the use of NSAIDs and proton pump inhibitors (PPIs). In particular, beta-blockers, ACE inhibitors/sartans and NSAIDs were associated with a risk of severe or fatal anaphylaxis [10,11^▪^,^12^].

## DRUGS

In Western countries, drugs are the first cause of anaphylaxis in the elderly and the prevalence of drug allergy, at this age, is between 0.6 and 2.1% [4]. Antibiotics and analgesics are the main classes of drugs involved in anaphylaxis at this age (over 65 years) [4,5,14^▪^]. Other classes of drugs less commonly involved are chemotherapeutics and contrast media [14^▪^].

In older adults, several studies have shown a correlation between polytherapy due to comorbidities and drug-induced anaphylaxis, and one of the causes could be represented by inappropriate drug prescription, which may be more likely to occur in this population group [15,16].

Moreover, Arroyo *et al.*[17] highlighted that drug-related anaphylaxis is the first cause of visits to emergency medicine departments and hospitalizations in the USA. Particularly, age over 65 years, pulmonary comorbidities (e.g., asthma) and cardiac diseases (e.g., coronary disease, heart failure) were reported as potential risk factors for severe or fatal drug-induced anaphylaxis [17,18].

### Insect stings

Stinging insect's venom is a major cause of anaphylaxis among pediatric and adult population [2]. Although insect allergy is more common in young adults, fatal anaphylaxis caused by insect stings is more likely to occur in older adults [2]. In Western countries, the most common venom triggers include *Hymenoptera* insects, as yellow jacket, hornet, wasp and honeybee [2,4].

Venom immunotherapy (VIT) is the most important treatment for *Hymenoptera* venom allergy, thanks to the induction of immune-tolerance granting from the risk of severe reactions in case of further stings. Of note, age does not affect the indication for VIT [19].

The clinical reactivity to insect venoms increases with age. Although the individual exposure to insect stings might be comparable among adults, one could speculate that retired elderly spend more time outdoor activities, that is, gardening. Another reason for increased reactivity might be the higher frequency of comorbidities, which may influence the clinical response patterns, particularly in patients with preexisting cardiovascular diseases, which are more prone to experience severe cardiovascular symptoms like tachyarrhythmia or syncope.

The main risk factors for severe or fatal anaphylaxis due to *Hymenoptera* stings are older age, cardiovascular disease and mastocytosis [2,4,19,20]. Contrarily to what already stated for food triggers [11^▪^,^12^], beta-blockers and ACE-inhibitors are not involved in more severe or fatal anaphylaxis due to *Hymenoptera* allergy, and they are not a contraindication to immunotherapy [4,19,21,22].

## CLINICAL PRESENTATION AND MANAGEMENT

Skin is usually the most frequently involved system in anaphylaxis but the occurrence of skin symptoms in the elderly is significantly reduced and mostly associated with severe anaphylaxis [4,6]. This could be explained by reduced cutaneous vascularization, and increased use of drugs such as beta-blockers or neuroleptics in this group of patients [6]. The occurrence of cardiovascular symptoms tends to increase linearly with age, while the frequency of respiratory symptoms decreases with advancing age [6]. The European Anaphylaxis Registry points out not only a correspondence of the severity of anaphylactic reactions with age, but also a more frequent concomitance of cardiovascular symptoms as well [6,23^▪▪^]. Additionally, several studies have shown higher rates of hospital and intensive care unit admissions in older adults [6,17,23^▪▪^,^24^].

Adrenalin is a life-saving therapy, and it represents the most important drug in anaphylaxis treatment, which is recommended as the first step in treatment by several guidelines [1,7].

Even though anaphylaxis in the elderly more often requires the administration of adrenaline, its use remains suboptimal [17,18]. Cardiovascular diseases and drugs needed to manage them (ACE-inhibitors, beta-blockers) are important factors making complex and challenging the treatment of anaphylaxis in older adults. Moreover, they may explain the low rates of adrenalin administration [17,18]. It is important to highlight that there is no absolute contraindication, in any age group, for the administration of adrenalin in the treatment of anaphylaxis [4,6,17,18,23^▪▪^,^24^].

## DISCUSSION AND CONCLUSION

Anaphylaxis is a potentially life-threatening allergic reaction that can occur at any age, and due to the higher prevalence of older adults because of the longer life expectancy, it shows an increase among the geriatric population [3^▪▪^,^4^]. The most common triggers of allergic reaction are drugs, venom and foods [4,6,17,18,23^▪▪^,^24^]. In addition, idiopathic anaphylaxis is seen more frequently in adults and older adults [23^▪▪^].

Elderly patients show a greater risk for severe or fatal reactions, because they often report multiple comorbidities (including cardiovascular diseases) involving the concomitant use of multiple therapies that may interfere with anaphylaxis management, thus leading to poor outcomes [6,17,18,23^▪▪^,^24^]. These factors are directly linked to higher rate of hospitalization and admission to intensive care [17,18].

Drug allergy, the first cause of anaphylaxis in the elderly, is particularly relevant in this age group because of the frequent polytherapy and, therefore, the higher probability to develop an allergy. Antibiotics and analgesics are the main classes of drugs involved in anaphylaxis in geriatric age [4,5,14^▪^,^23^^▪▪^]. On the other hand, polypharmacy may make the identification of the culprit drug more difficult and complex, especially when it is a pivotal drug that cannot be easily withdrawn.

The second cause of anaphylaxis in the older adults is venom [2,4,23^▪▪^]. In Europe the most common triggers include *Hymenoptera,* and, in this subset of patients, the main risk factors for severe or fatal anaphylaxis include older age, cardiovascular disease and mastocytosis [2,4,19,20]. Notably, VIT remains the only disease-modifying treatment for *Hymenoptera* venom allergy at any age [19].

Although food allergy is less commonly a cause of anaphylaxis in the elderly, aging is accompanied by several physiological and pathological changes that can mask food allergy symptoms or promote them, as gastrointestinal, pulmonary and cardiovascular comorbidities, immunosenescence, augmented inflammatory status, microbiota alterations, and micronutrient deficiency [3^▪▪^,^4^]. The patterns of FIA seem to be quite different in older people compared to younger groups., with seafood, mammalian meat, legumes, fruits and vegetables as most frequent elicitors. Interestingly, even PFAS, generally known as a low-risk condition, is described as one of the causes of FIA with geriatric-onset, due to age-related factors affecting mastication and quality of saliva, atrophic gastritis, use of antiulcer drugs and increased gastrointestinal inflammation promoting intestinal permeability [3^▪▪^,^10^,^12^].

The clinical presentation of anaphylaxis in the older adults is most often characterized by cardiovascular involvement, with syncope as the most frequent symptom [4,6]. The occurrence of skin and respiratory symptoms is characterized by an inverse relationship with age, resulting significantly lower in the elderly compared to younger populations [6]. Moreover, their involvement is often associated with severe anaphylaxis [4,6]. Aside from the specific clinical manifestations, several authors highlighted that age over 65 years is an important risk factor for severe or fatal anaphylaxis [4,6,17,18,23^▪▪^,^24^].

As reported in the current guidelines, the injection of adrenaline is life-saving and remains the most important treatment of anaphylaxis at any age [1,7], but its use is still suboptimal. However, in case of anaphylaxis, no absolute contraindications for the older age are reported [4,6,17,18,23^▪▪^,^24^].

In light of the above and considering the increasing life-expectancy of population, especially in the Western areas, greater attention should be addressed on this topic in coming years, in order to achieve more useful information to define and manage anaphylaxis in such a delicate phase of life which is old age.

## Acknowledgements

*None.*

### Financial support and sponsorship

*This review had no funding.*

### Conflicts of interest

*A.B., F.N., M.O. and E.R. declare no conflicts of interest*

## REFERENCES AND RECOMMENDED READING

Papers of particular interest, published within the annual period of review, have been highlighted as:

▪ of special interest
▪▪ of outstanding interest

## References

[1] Cardona V Ansotegui IJ Ebisawa M. World allergy organization anaphylaxis guidance 2020. World Allergy Organ J 2020; 13:100472. 33204386 10.1016/j.waojou.2020.100472 PMC7607509

[2] Dribin TE Motosue MS Campbell RL. Overview of allergy and anaphylaxis. Emerg Med Clin North Am 2022; 40:1–17. 34782082 10.1016/j.emc.2021.08.007 PMC8604419

[3▪▪] Azzolino D Verdi L Perna S. Food allergies in older people: an emerging health problem. World Allergy Organ J 2024; 17:100967. 39310373 10.1016/j.waojou.2024.100967 PMC11416488

[4] Ventura MT Boni E Taborda-Barata L. Anaphylaxis in elderly people. Curr Opin Allergy Clin Immunol 2022; 22:435–440. 36165408 10.1097/ACI.0000000000000855 PMC10815006

[5] Martins-Dos-Santos G Araújo M Prates S Leiria-Pinto P. Immunoallergic disorders in the elderly. Eur Ann Allergy Clin Immunol 2022; 54:175–182. 33949173 10.23822/EurAnnACI.1764-1489.211

[6] Aurich S Dölle-Bierke S Francuzik W. Anaphylaxis in elderly patients-data from the European Anaphylaxis Registry. Front Immunol 2019; 10:750. 31068925 10.3389/fimmu.2019.00750 PMC6491699

[7] Muraro A Worm M Alviani C. EAACI guidelines: anaphylaxis (2021 update). Allergy 2022; 77:357–377. 34343358 10.1111/all.15032

[8] Warren C Nimmagadda SR Gupta R Levin M. The epidemiology of food allergy in adults. Ann Allergy Asthma Immunol 2023; 130:276–287. 36509408 10.1016/j.anai.2022.11.026

[9] Sampath V Abrams EM Adlou B. Food allergy across the globe. J Allergy Clin Immunol 2021; 148:1347–1364. 34872649 10.1016/j.jaci.2021.10.018

[10] Hultquist H Dyer A Jiang J. Phenotypic characterization of childhood- and adult-onset food allergy among adults in the United States. J Allergy Clin Immunol Glob 2022; 1:257–264. 36425303 10.1016/j.jacig.2022.05.011 PMC9683432

[11▪] El Hanache H Perennec T Beaumont P. Food anaphylaxis in the elderly: analysis of allergy vigilance network data from 2002 to 2021. Clin Exp Allergy 2023; 53:561–572. 36811252 10.1111/cea.14297

[12] Boyle RJ Shamji MH. Food anaphylaxis in older people. Clin Exp Allergy 2023; 53:488–490. 37154015 10.1111/cea.14330

[13] Asero R Ariano R Aruanno A. Systemic allergic reactions induced by labile plant-food allergens: seeking potential cofactors. A multicenter study. Allergy 2021; 76:1473–1479. 33080053 10.1111/all.14634

[14▪] Butranova O Zyryanov S Gorbacheva A. Drug-induced anaphylaxis: National Database Analysis. Pharmaceuticals (Basel) 2024; 17:90. 38256923 10.3390/ph17010090 PMC10821106

[15] O’Mahony D Gudmundsson A Soiza RL. Prevention of adverse drug reaction in hospitalized older patients with multimorbidity and polypharmacy: the SENATOR* randomized controlled clinical trial. Age Ageing 2020; 49:605–614. 32484850 10.1093/ageing/afaa072

[16] Hedna K Hakkarainen KM Gyllensten H. Potentially inappropriate prescribing and adverse drug reactions in the elderly: a population based study. Eur J Clin Pharmacol 2015; 71:1525–1533. 26407684 10.1007/s00228-015-1950-8 PMC4643104

[17] Arroyo AC Robinson LB Cash RE. Trends in emergency department visits and hospitalizations for acute allergic reactions and anaphylaxis among US older adults: 2006-2014. J Allergy Clin Immunol Pract 2021; 9:2831–2843. e8. 33798790 10.1016/j.jaip.2021.03.032 PMC8277683

[18] Meir LR, Habbsa S, Waqar O, *et al*. Anaphylaxis among elderly emergency department patients in a large health system in New York. Ann Allergy Asthma Immunol 2022; 129:63-70.e3, ISSN 1081-1206. 10.1016/j.anai.2022.03.020. 35346881

[19] Sturm GJ Varga EM Roberts G. EAACI guidelines on allergen immunotherapy: hymenoptera venom allergy. Allergy 2018; 73:744–764. 28748641 10.1111/all.13262

[20] Pawłowicz R Bożek A Dor-Wojnarowska A. Elderly patients and insect venom allergy: are the clinical pictures and immunological parameters of venom allergy age-dependent? Vaccines (Basel) 2024; 12:394. 38675776 10.3390/vaccines12040394 PMC11053896

[21] Kaakati RN Khokhar D Akin C. Demographics, types of patient-reported allergic diseases, and anaphylaxis in mastocytosis: a single-center US experience. J Allergy Clin Immunol Pract 2025; 13:398–406. 39515518 10.1016/j.jaip.2024.10.039

[22] Golden DBK Wang J Waserman S. Anaphylaxis: a 2023 practice parameter update. Ann Allergy Asthma Immunol 2024; 132:124–176. 38108678 10.1016/j.anai.2023.09.015

[23▪▪] Yildiz E Arslan Ş Çölkesen F. Anaphylaxis in older adult patients: a 10-year retrospective experience. World Allergy Organ J 2022; 15:100665. 35891674 10.1016/j.waojou.2022.100665 PMC9293944

[24] Arroyo AC Camargo CA. The importance of understanding anaphylaxis among older adults. Ann Allergy Asthma Immunol 2022; 129:7–8. 35717136 10.1016/j.anai.2022.04.024 PMC9639602

